# Benefits and Limitations of OCT-A in the Diagnosis and Follow-Up of Posterior Intraocular Inflammation in Current Clinical Practice: A Valuable Tool or a Deceiver?

**DOI:** 10.3390/diagnostics12102384

**Published:** 2022-09-30

**Authors:** Carl P. Herbort, Ioannis Papasavvas, Ilknur Tugal-Tutkun

**Affiliations:** 1 Retinal and Inflammatory Eye Disorders, Centre for Ophthalmic Specialised Care (COS), 1003 Lausanne, Switzerland; 2 Department of Ophthalmology, Istanbul Faculty of Medicine, Istanbul University, Istanbul 34093, Turkey

**Keywords:** OCT-A, FA, ICGA, posterior uveitis, MFC, APMPPE, serpiginous choroiditis, MEWDS, Behçet uveitis, birdshot chorioretinopathy, VKH

## Abstract

Purpose: Optical coherence tomography angiography (OCT-A) has been applied to uveitis and intraocular inflammation since its availability after 2014. The imaging of retinal and choroidal vascularization without the use of dyes was a major development and represented a potentially valuable tool in ocular research. In addition to such use, OCT-A is often put forward as being able to potentially replace invasive methods needing dye injection, such as fluorescein angiography (FA) and indocyanine green angiography (ICGA). The aim of this review was to establish whether OCT-A was sufficiently useful in everyday routine clinical practice to monitor disease evolution and to perform treatment adjustments to the extent that it could reliably replace the standard dye methods. Methods: Selective literature review and analysis of own data and experience. Results: OCT-A is a technologically high-grade imaging modality allowing to analyze retinal circulation in inflammatory diseases of the posterior pole with a high sensitivity useful for research purposes. However, there is no evidence that it reaches equal effectiveness in the routine management of posterior uveitis involving the retina. OCT-A is unable to show leakage. In choriocapillaritis involving pre-capillary vessels, it shows capillary drop-out but does not seem to have an advantage over ICGA except that it can be repeated easily, not being invasive, and so allows a closer follow-up. It is, however, less useful in end-choriocapillary non-perfusion, such as in MEWDS. For choroidal stromal inflammation, OCT-A is ill-suited as it only shows inconsistent secondary circulatory changes produced by choroidal foci. OCT-A seems to be useful in the diagnosis and follow-up of inflammatory chorioneovascularisation (iCNV), although dye exams are more precise in showing the activity of the iCNV. Conclusion: In summary, OCT-A is a very sensitive modality for the retinal circulation in uveitis for research purposes; it is sometimes useful for close follow of choriocapillary drop-out but not in end-capillary non-perfusion. Its use for monitoring purposes in stromal choroiditis, however, is questionable. Its claim to possibly replace classical angiographic work-up for the practical management of posterior uveitis is largely overrated.

## 1. Introduction

There has been significant development of ocular imaging modalities since the mid-1990ties that have progressively found their respective domains of application in uveitis. Unfortunately, the error often lies in overestimating the capabilities that such new modalities bring. Moreover, understandably, the trend to try to replace invasive imaging methods with non-invasive methods strongly prevails nowadays and represents a strong incentive to favor non-invasive methods even though these methods are sometimes far from being satisfactory and only partially replace or completely fail to replace more global standardized modalities in current uveitis practice.

Optical coherence tomography angiography (OCT-A) does not escape this pitfall. Even more, it is technically a very elaborate modality leading to an overestimation of its utility in uveitis common practice in general and in non-infectious choroiditis in particular. Consequently, numerous publications were performed applying OCT-A to uveitis and/or non-infectious choroiditis, very often generating inconsistent and inconclusive data as far as monitoring utility is concerned.

OCT-A is a relatively recent non-invasive imaging method that demonstrates retinal and choroidal blood flow without the use of fluorescence dyes. The mechanism through which OCT-A outlines vasculature is based on the detection of motion of blood cells, especially red blood cells in the vessels, analyzing the different reflections of multiple chronological OCTs [1]. It is actually used in the diagnosis and follow-up of age-related macular degeneration, retinal vascular diseases such as diabetic maculopathy, retinal artery/vein occlusion, and other entities that produce retinal perfusion alterations [2]. In an attempted pioneering pragmatism, OCT-A was reported to supposedly have a role in monitoring posterior uveitis, namely in the retinal vascular pathology of uveitis [3]. The technology is a valuable addition to investigation of intraocular circulation and is at the origin of progress in research in chorioretinitis. The major problem, however, is that the narrative of most studies on OCT-A in uveitis addresses the possible replacement by OCT-A of historic well-established methods proven to be reliable and global in the practical management of posterior uveitis [4]. Indeed, in most OCT-A studies reported, the data generated correspond to research results rather than to practical management utility. 

The aim of this review and perspective was to evaluate whether there are situations in which OCT-A might show some potential practical use in common uveitis practice or whether this is not the case. To accomplish this, we analyzed several current uveitis entities and made a critical review of the role of OCT-A regarding its potential to replace established methods in patient management in common uveitis practice.

## 2. OCT-A Technology

### Technical Details of OCT-A and Image Processing

OCT-A is an evolution from OCT technology. Actually, it uses light reflectance of the movement of red blood cells, which are moving inside a vessel, through multiple repeated scans (A-scans) of an area. These images are analyzed, and differences are detected between scans revealing zones with high/low flow or no flow vessels [5]. There are different techniques used to quantify these changes, using full-spectrum or split spectrum [6,7,8,9]. The latter, described by Jia et al. in 2012 [10], significantly minimizes motion noise with better visualization of the vessels. Light is emitted through either a spectral domain OCT (SD-OCT) having a wavelength of 800 nm or a swept-source OCT (SS-OCT) with a wavelength close to 1050 nm (Table 1). The difference is that longer wavelengths have a deeper tissue penetrance, but they also present slightly lower axial resolution.

Until now, OCT-A has had a limited field of view with images restricted to the posterior pole, a limitation that reduces its usefulness in the practical management of inflammatory diseases that, in most cases, affect predominantly the peripheral areas of the fundus in addition to the posterior pole. However, SS-OCT and SS-OCTA wide field modules such as the Xephilio OCT-S1 from Canon^®^ are already available, which allow single 20 × 23 mm OCTA scans, which can be expanded to 31 × 27mm using an optional mosaic software capturing the anterior edge of all four vortex veins. Images created by OCT-A montage are often used to compare with FA-ICGA, but the creation of this montage has many technical difficulties and, in the majority of cases, needs special software. As it is mentioned by Eastline et al., their study showed that 100% of montage-wide field OCTA images present artifacts, including but not limited to displacement-, shadowing- and artifact and vessel displacement [11,12]. Hypopyon, vitritis, and exudative detachment of the retina can also affect the quality of OCT-A

Furthermore, practical aspects have to be taken into account, such as the fact that OCT-A should be obtained with devices accessible to most clinical centers rather than with expensive research instruments to which only research centers can have access with restricted access for everyday practice

## 3. Use of OCT-A in Clinical Practice for Specific Uveitis Conditions

### 3.1. Retinal Inflammatory Diseases

#### 3.1.1. Behçet Uveitis

Behçet’s uveitis is a panuveitis with typical ocular findings such as non-granulomatous anterior uveitis, hypopyon, vitritis, retinal vasculitis, retinal infiltrates, and papillitis [13]. Retinal vasculitis of the posterior segment is a prominent feature of Behçet’s uveitis, and FA is the gold standard investigation to detect leakage from vessels. 

**Figure 1:**
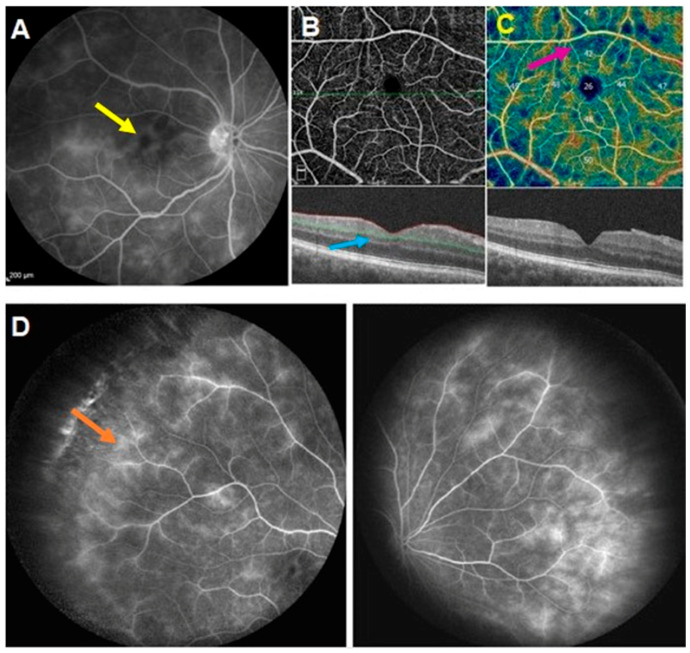
Behcet Uveitis, same-day imaging. (**A**) Fluoresceine angiography of the posterior pole showing macular edema (yellow arrow). (**B**): en face (**top**) and cross-sectional (**bottom**) OCT-A scans of the same patient. Macular edema is not detected by the cross-sectional scan (blue arrow). (**C**) vascular density showing defects of the superficial plexus (Grimson arrow). (**D**) Fluoresceine of the periphery showing mild vasculitis (orange arrow).

OCT-A is showing vessels but is unable to show leakage, which is a crucial factor in assessing and following the disease and which, therefore, represents a serious limitation of OCT-A in Behçet’s uveitis (Figure 1). Moreover, as for most diseases, the limited field of view in a disease with diffuse vascular involvement hampers its use for routine monitoring of Behçet’s uveitis. However, OCT-A was shown to provide complementary information by demonstrating secondary complications of vasculitis such as macular capillary drop-out, increased Foveal Avascular Zone (FAZ), and vascular abnormalities, including shunts and telangiectasias (Figure 1C) [14]. 

Khairallah et al. compared FA and OCT-A findings in patients with Behçet’s uveitis and concluded that OCT-A visualizes and characterizes perifoveal microvascular changes better than FA in eyes with active Behçet’s uveitis. They also illustrate a correlation between posterior pole ischemia on FA and OCT-A findings, such as the presence of perifoveal capillary arcade disruption in the superficial capillary plexus (SCP) greyish nonperfusion/hypoperfusion areas in both the superficial and the deep capillary plexuses (DCP) as well as capillary network disorganization in both the superficial and the deep capillary plexuses [15]. Although these findings are interesting, it is doubtful that they will contribute practically to the monitoring, follow-up, and adjustment of therapy. In contrast to posterior pole findings, these authors did not find any correlation between the presence of peripheral retinal ischemia on FA and any of the OCTA pathologic features. As a result, FA should still be considered the gold standard imaging modality in detecting and evaluating peripheral vasculitis and neovascularization.

#### 3.1.2. HLA-A29 Birdshot Retinochoroiditis (BRC)

HLA-A29 birdshot retinochoroiditis is an autoimmune inflammatory ocular disease including dual simultaneous involvement of the retina in the form of a pronounced retinal vasculitis of vessels of all sizes and of the choroid in the form of a stromal choroiditis. The disease, including its retinal involvement, is discussed in the paragraph on stromal choroiditis. 

### 3.2. Choriocapillaritis Entities

#### 3.2.1. Multiple Evanescent White Dot Syndrome (MEWDS)

**Figure 2:**
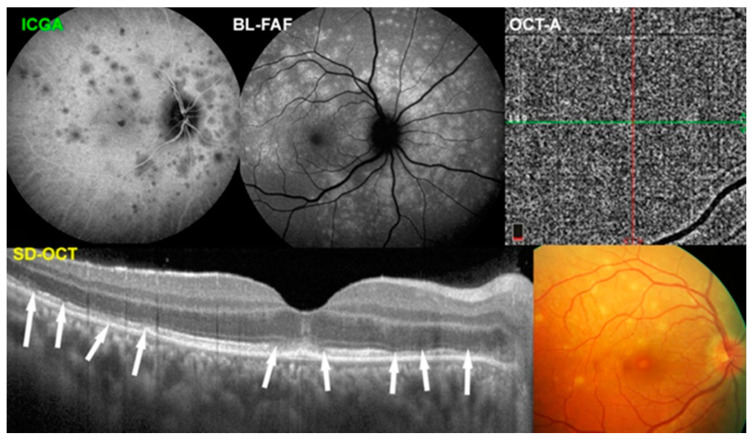
Multimodal imaging in MEWDS. End-choriocapillary non-perfusion is clearly demonstrated on ICGA (**top left**), corresponding to areas delineated by BL-FAF (**top middle**) and to the fundus lesions (**bottom right**). OCT-A does not show any capillary drop-out as it is not able to image low flow end-choriocapillary circulation (**top right**). SD-OCT (**bottom left**) shows loss of photoreceptor outer segments (white arrows) corresponding to ICGA and BL-FAF lesions.

Multiple evanescent white dot syndrome is a primary choriocapillaritis situated at the benign end of the spectrum [16]. It is unilateral disease characterized by pale dots in the posterior fundus and mid-periphery seen only in the early phase of the disease. Often, when patients consult because of photopsias and subjective scotomas, the lesions are no more visible, but the central macula may display a granular aspect [17]. Physiopathologically, the disease is produced by distal end-choriocapillary non-perfusion, which explains the patchy, scattered, often non-confluent morphology of lesions. The gold standard to precisely identify the localization and extent of non-perfusion is ICGA [18,19]. Because of limited ischemia, the retinal pigment epithelium, a metabolically resistant structure, is not damaged in most cases, while the photoreceptor outer segments, metabolically very demanding, are damaged as evidenced on SD-OCT showing loss of photoreceptor outer segments, which is corroborated by blue-light fundus autofluorescence showing hyperautofluorescence (Figure 2). OCT-A is of limited use in end-capillary vessels; it is only minimal or no flow, the latter being necessary for OCT-A to demonstrate vessels. The choriocapillaris, therefore, appears to be normal (Figure 2). This is the reason why MEWDS has falsely been assimilated into primary photoreceptoritis by some authors [20,21]. The same phenomenon applies to mild or to the initial episode of MFC presenting the same pathophysiological process. Indeed, the first episode of MFC can present itself as MEWDS before the diagnosis has to be revised when recurrences and scars occur [22].

#### 3.2.2. Idiopathic Multifocal Choroiditis (MFC)

**Figure 3:**
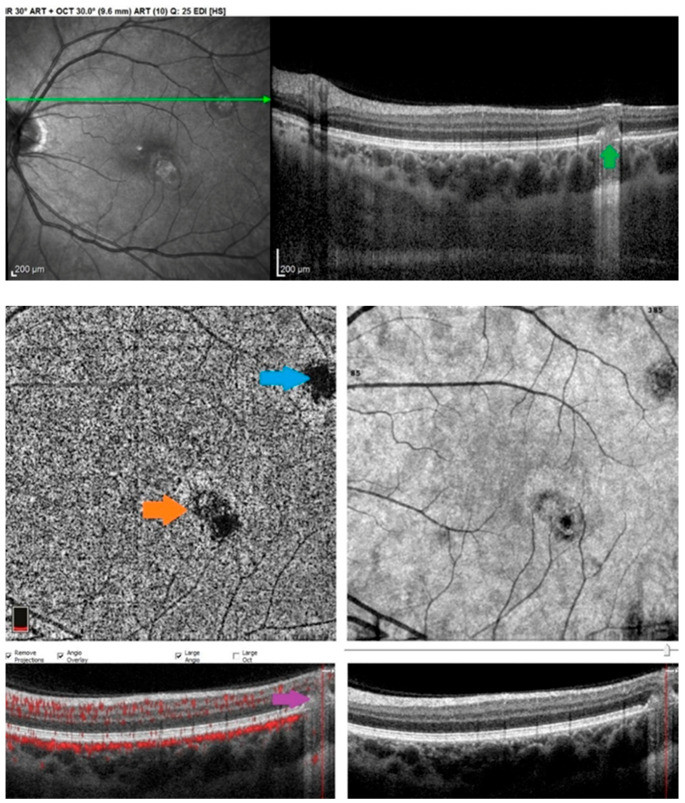
Inability of OCT-A to show active versus inactive choroidal neovessels. Upper image: OCT cross-sectional scan of an active MFC lesion (green arrow), which presents as a dark spot in choriocapillaris en-face OCTA scan (blue arrow, **middle left**) due to choriocapillaris drop-out, as well as absence of flow in cross-sectional scan (Crimson arrow, **lower left**). The orange arrow pinpoints a dark lesion with a flow detection inside, corresponding to iCNV, which in this case was inactive.

Idiopathic Multifocal Choroiditis (MFC) is a primary choriocapillaritis for which the trigger is unknown as for most entities of this group. The first episode, when no chorioretinal scars are present yet, can look like MEWDS. In these cases, the diagnosis is revised when recurrences with scars occur. On top of end-choriocapillary involvement, non-perfusion is prolonged and can also affect larger vessels, which explains the development of chorioretinal scars. ICGA hypofluorescent areas delineate very precisely the localization and extent of non-perfused areas, co-localizing with BL-FAF hyperautofluorescent areas. The latter corresponds to the loss of photoreceptor outer segments shown on SD-OCT, which gives better visual access to fluorophores normally present in the pigment epithelium. OCT-A may be negative in end-choriocapillary non-perfusion because of low flow or absence of flow, such as in MEWDS. In areas where larger choriocapillaris vessels are involved, OCT-A can show choriocapillaris drop-out [23]. MFC often presents not only with widespread occult non-perfused areas only seen on ICGA but also much more prolonged non-perfusion than MEWDS. This explains the much higher rate of inflammatory choroidal neovascularization (iCNV), which can be a complication in close to 30% of cases. OCT-A is useful in the detection and follow-up of iCNV in patients with MFC but can also help differentiate an active inflammatory lesion from an iCNV in the absence of dye exams [24]. Active MFC lesions of sufficient extension present as dark choriocapillaris areas on OCT-A en-face scans due to nonperfusion (choriocapillary drop-out), whereas iCNVs present as a high-flow bright network of vessels (Figure 3). On the cross-sectional scans, iCNVs present with a flow signal inside the lesion; in contrast, there is no flow signal inside an active MFC lesion. Compared to FA and ICGA, OCT-A cannot distinguish active iCNVs from inactive iCNVs, both appearing as high-flow bright networks. Indeed, Cheng et al. reported that 60% of inactive iCNV in MFC patients presented blood flow on OCT-A, while other modalities clearly showed that iCNVs were inactive [25]. This incapacity of OCT-A to demonstrate leakage is a shortcoming of OCT-A that must be taken into account by the specialist when interpreting an OCT-A image of iCNV in cases of MFC. Consequently, ICGA and FA remain the best imaging modality to delineate choriocapillaris non-perfusion and to demonstrate active iCNVs. As for most uveitis entities, the major shortcoming of OCT-A is, however, its limited field of view in diseases that involve the whole fundus.

#### 3.2.3. Acute Posterior Multifocal Placoid Pigment Epitheliopathy/Acute Multifocal Ischaemic Choriocapillaritis (APMPPE/AMIC)

**Figure 4:**
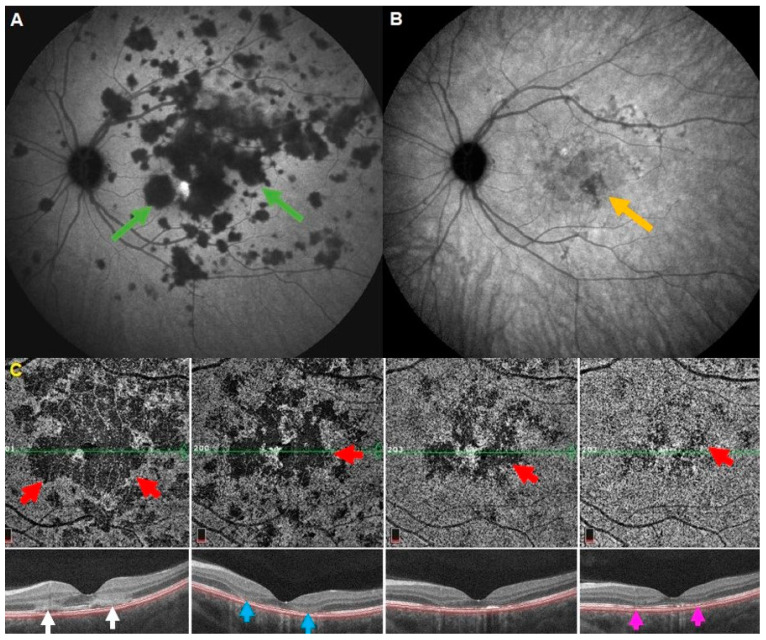
**Imaging of a patient with APMPPE, ICGA vs. OCT-A.** (**A**), Late frame of ICGA at presentation where non perfusion of choriocapillaris is presented as hypofluorescent areas (green arrows). (**B**), late frame of ICGA after treatment where choriocapillaris non perfusion is significantly reduced, leaving some scars (orange arrow). (**C**), Evolution of the OCT-A from presentation (**left** frame) to last control (**right** frame). Red arrows demonstrating choriocapillaris drop-out and the favorable evolution after treatment. Cross-sectional scans (**bottom** images) are showing hyperreflective lesions of outer retina (white arrows), which evolutes to outer segment loss (blue arrows) and restoration of OS in last control (Grimson arrows). OCT-A helped to follow up the evolution of the patient between the two dye exams but was restricted to posterior pole.

Acute posterior multifocal placoid pigment epitheliopathy, also better called acute multifocal ischaemic choriocapillaritis (APMPPE/AMIC), is a primary choriocapillaritis with very diverse severity going from cases with minimal areas of non-perfusion to very severe ischemia when larger choriocapillaris or pre-choriocapillaris vessels are involved. The choriocapillaris non-perfusion caused by APMPPE/AMIC is demonstrated in a detailed fashion by ICGA (Figure 4A,B). OCT-A en-face scans show the same image of choriocapillary non-perfused areas (choriocapillary drop-out) as ICGA in the limited posterior pole area analyzed by OCT-A (Figure 4C) [26]. Choriocapillaris non-perfusion causes substantial ischemia of the outer retina visualized as hyperreflective lesions in the cross-sectional scans (Figure 4C, white arrows). In cases with limited severity, these alterations are reversible, while in more pronounced cases, immunosuppressive treatment is needed [27]. The OCT-A findings within the 6 × 6 mm window analyzed correlate well with the hypofluorescent zones demonstrated by ICGA. Furthermore, outer retinal alterations on SD-OCT co-localize with the zones of choriocapillaris drop-out on OCT-A [28,29]. OCT-A is useful as part of the multimodal imaging in the follow-up of APMPPE within its limited field of view and can play a more important monitoring role because it can be repeated easily as it is non-invasive. Dark areas can represent either active lesions (choriocapillaris drop-out) or chorioretinal scars.

#### 3.2.4. Serpiginous Choroiditis (SC)

**Figure 5:**
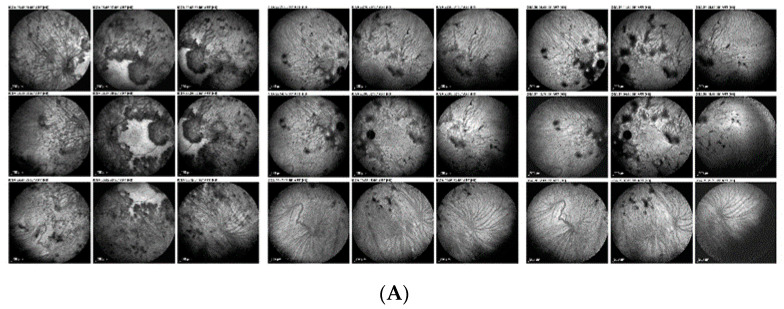

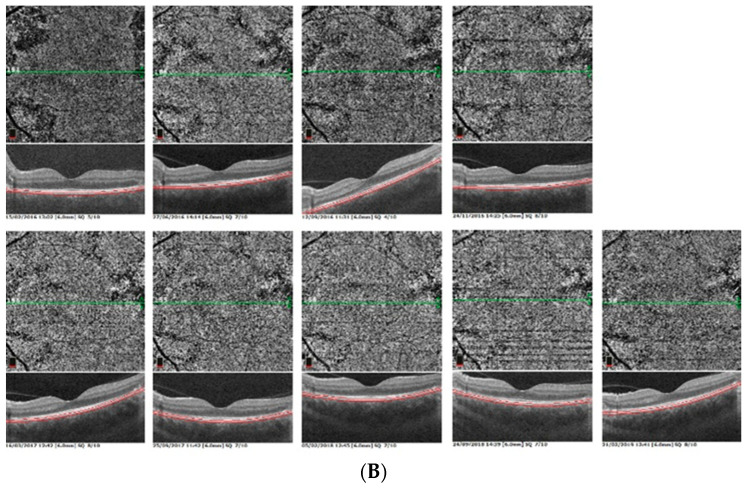
Tuberculosis-related SC. ICGA versus OCT-A. This patient’s lesions were mostly outside the macula. Therefore, ICGA (**A**) was much more precise for follow-up and treatment monitoring (**A**), whereas OCT-A (**B**) only showed evolution of the rare central lesions (**B**).

Serpiginous choroiditis (SC) is situated on the severe side of the choriocapillaritis spectrum involving larger choriocapillaris and pre-choriocapillaris vessels, causing extended and progressing areas of non-perfusion. It is either idiopathic or can develop in patients that have been in contact with Mycobacterium tuberculosis objectivized by performing an interferon gamma release assay (IGRA/QuantiFERON Gold test). Both conditions will be discussed together. ICGA has been shown to be the most reliable imaging modality to globally identify the extent of lesions and monitor the evolution of the disease and the response to therapy [28]. OCT-A was shown to supplement other imaging techniques, such as ICGA and EDI-OCT [30]. It helped in the comprehension of the leading changes of the choroidal vascular network in active and inactive lesions of SC [31]. An interesting study showed the different evolution of the choriocapillaris in large and small SC lesions after healing. The large lesions evolved to choriocapillaris atrophy while small lesions evolved to resolution of choriocapillaris non-perfusion [32]. Perfusion density, choroidal vascularity index, and choriocapillaris flow deficit were shown to be significantly lower in SC areas than in normal areas giving a finer insight into choriocapillaris pathology in SC [33]. The numerous recent studies contributed greatly to the research of the choriocapillaris in SC. However, studies only rarely suggested that OCT-A might play the role of a monitoring device, and those that hinted at this possibility indicated that further research was needed to standardize parameters to aid in uniform management and follow-up of cases [34]. In our hands, ICGA was still irreplaceable for global and precise assessment of lesions, often going beyond the vascular arcades, as well as for their follow-up and for monitoring the efficacy of therapeutical intervention, as shown in Figure 5 [35]. We also found that ICGA was more precise than OCT-A in showing subclinical lesions in SC [36]. Although OCT-A will not replace ICGA totally because of the limited field of view, it will contribute to diminishing ICGA, as central lesions can more often be checked, as the method is non-invasive (Figure 5).

### 3.3. Stromal Choroiditis Entities

#### 3.3.1. Ocular Sarcoidosis (OS)

Sarcoidosis is a systemic disease that affects the eye, among other organs. Ocular manifestations of sarcoidosis chorioretinitis include bilateral granulomatous anterior uveitis, vitritis, sometimes with the presence of snowballs, periphlebitis, choroidal stromal foci presenting as multifocal choroiditis, retinal microaneurysms, macular and optic disc edema. As in Behçet’s uveitis, vascular pathologic findings are described on OCT-A. Cerquaglia et al. presented the utility of OCT-A in demonstrating perifoveal vascular alterations, such as hypo-perfused/non-perfused areas in the SCP and DCP, capillary abnormalities, and capillary network disorganization, in patients with ocular sarcoidosis (OS). It was further noteworthy that DCP was more affected than SCP in patients with OS [37]. Similar to studies on Behçet’s uveitis, OCT-A was obviously not capable of detecting neither peripheral retinal ischemia nor leakage.

As far as OCT-A imaging of choriocapillaris in patients with OS is concerned, Pichi et al. reported that larger, full thickness choroidal granulomas can be visualized on OCT-A as areas of choriocapillaris drop-out that co-localized with the ICG hypofluorescent lesions [3].

Other case reports have also mentioned choriocapillaris non perfusion dark areas in OCT-A en-face scans of the posterior pole [38]. 

Such anecdotal reports give interesting insights into the possibilities of additional imaging gained by OCT-A, but they, by no means hint that OCT-A could contribute significantly to the practical management of such a global eye disease involving the central and peripheral retina and the choroid. In contrast, FA and ICGA were shown to give a quantitative and global evaluation of the proportional involvement of the retinal and choroidal compartment and their precise evolution [39].

#### 3.3.2. HLA-A29 Birdshot Retinochoroiditis (BRC)

HLA-A29 birdshot retinochoroiditis (BRC) is an autoimmune inflammatory disease linked to the presence of the human leucocyte antigen A-29 (HLA-A29), which is involved in parallel but independently of the retina and the choroid, meaning that inflammation in one compartment (retina) is not the consequence of inflammation in the other (choroid) and vice versa [40]. The gold standard to assess and monitor both compartments are respectively FA for the retina and ICGA for the choroid [41]. Since OCT-A has become available, there has been an almost irresistible trend to impose OCT-A as a viable alternative to FA and ICGA [4]. This is natural and comprehensible as OCT-A is a non-invasive and easily repeatable imaging modality. There is no doubt that OCT-A is an interesting research tool for exploring disease mechanisms. The question, however, is whether the proven, accurate, and well-standardized FA and ICGA giving precise and global information on retina and choroid should or could be replaced by a potentially less performing or even insufficient modality for clinical monitoring because of the advantage of being non-invasive. The question of whether OCT-A can valuably replace FA and ICGA in BRC has to be dealt with separately for the retina and for the choroid, as the quality of information obtained by OCT-A seems to differ for the retina and for the choroid.

As far as the retina is concerned, several publications showed that OCT-A gives detailed information on the SPC and DPC of the retinal circulation [42,43]. One article showed that the capillary density of the full retina and the superficial and deep capillary plexuses were abnormal in BRC and linked to a decrease in visual acuity, which is an interesting finding [42]. The question, however, is whether this methodology will be useful for assessing and adapting treatment. Indeed, the evolution of such inflammatory parameters occurs slowly, and they have to be standardized to be of practical clinical use, whereas retinal vasculitis evaluated by FA, analyzed qualitatively or semi-quantitatively by using an angiographic scoring system [44] was shown to be of strong practical use to monitor disease and adapt treatment. Moreover, OCT-A does not give the global information that is available through FA as it is limited to the posterior pole.

**Figure 6:**
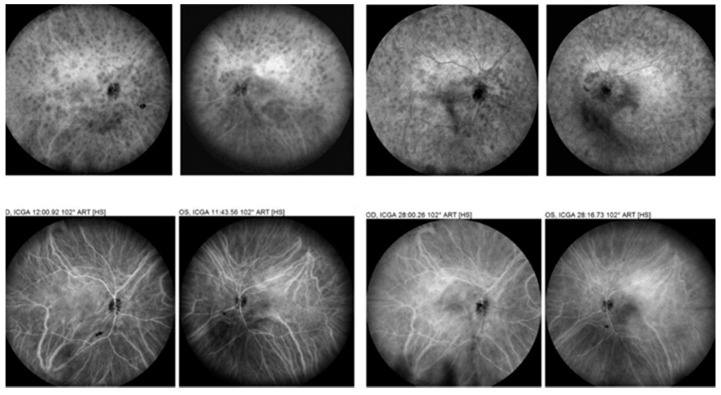
ICGA in HLA-A29 birdshot retinochoroiditis. Most of the stromal lesions are far beyond the field of view of the best OCT-A instruments, giving an excellent global well-defined view of the lesions not obtained otherwise.

As far as choroidal involvement is concerned, technically, the suitability of OCT-A is less obvious than its retinal utility in BRC. Indeed, it has been histologically proven that the active choroidal lesion process takes place in the mid-stroma and that the choriocapillaris and the pigment epithelium are not touched in the early stages of the disease [45]. So far, one study tried to show that OCT-A was able to show the same lesions as those seen on ICGA [46]. Contrary to what is indicated in this study, the lesions seem to be more precisely shown on ICGA (Figure 6). Moreover, these very preliminary choroidal results were obtained with a research instrument not commercially available. Further, images went up to a considerable 100° field, which cannot be obtained with common commercially available instruments. Despite these wide-field images that were obtained, the information is still less global than the imaging obtained with ICGA, as is shown in Figure 4.

Clearly, for BRC, OCT-A cannot be considered as a substitute for FA and ICGA, as far as the assessment of lesions, monitoring of disease evolution, and treatment adjustments are concerned. In the foreseeable future, this technology seems ill-suited for this pathology. It is, however, an interesting research tool prone to give some additional information on the macular retinal circulation and disease mechanisms of BRC.

#### 3.3.3. Vogt–Koyanagi–Harada (VKH)

**Figure 7:**
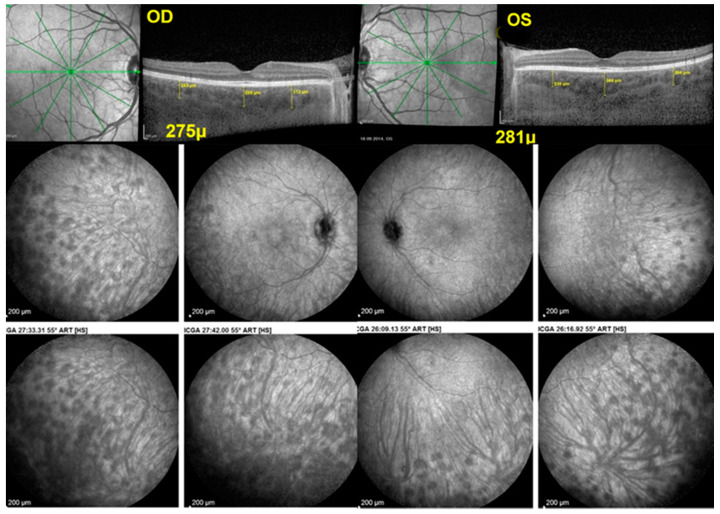
ICGA of Vogt–Koyanagi–Harada disease showing only peripheral lesions. After 6 months of triple immunosuppressive treatment, the patient still complained of headaches. FA and EDI-OCT were normal, and ICGA showed remaining lesions only present in the periphery out of the scope of OCT-A.

Vogt–Koyanagi–Harada disease (VKH) is a stromal choroiditis with full-thickness diffuse infiltration of the choroid by inflammatory cells [47]. It differs from BRC, which presents isolated stromal non-confluent foci. The other difference is that VKH is a strictly choroidal disease, and the retinal compartment is only involved secondarily when inflammation spills over from the choroid [48]. The established imaging modalities which directly show the choroidal involvement are, on one side, ICGA, which globally shows over the whole fundus the diseased areas in the choroid in the form of hypofluorescent dark dots [49], and on the other hand, enhanced depth imaging optical coherence tomography (EDI-OCT) that marks the diffuse thickening of the choroidal stroma in early disease and its subsequent evolution after treatment (Figure 7) [50].

OCT-A can only show the consequence of the choroidal infiltration on vascular flow but not directly the lesion process, giving, so to say, second hand non-systematized information. While OCT-A is very performing in analyzing retinal vessels in the posterior pole and can show choriocapillaris non-perfusion in some instances, it is ill-suited to analyze the choroidal stroma. It is, therefore, not surprising that there are so few publications on the use of OCT-A in the VKH [12,51]. Despite the inadequacy of the method for stromal choroiditis, rare reports tried to show the utility of OCT-A in the 6 × 6 posterior pole area analyzed by OCT-A [12,51]. Such findings were certainly interesting but cannot be systematized and, therefore, cannot be used for disease management, especially when knowing that the bulk of lesions in VKH is situated outside this posterior pole area (Figure 5). Such shortcomings of the method should be mentioned in OCT-A studies in order not to imply that VKH can be followed by OCT-A.

As a general observation, OCT-A studies in uveitis are mostly unsatisfactory as the unconscious or open purpose of the authors is to make the method look as if it could in future replace FA and ICGA. With this perspective in mind, an attempt was made to compare OCT-A to ICGA in posterior uveitis [12]. Methodologically the study was flawed for several reasons. Indeed, it is inadequate to perform such a correlation using a potpourri of posterior uveitis conditions. Both ICGA and OCT-A represent lesions having different pathophysiological explanations from one disease to another, going from perfusion disturbance to choroidal foci to chorioretinal atrophy. If a comparison were to be undertaken, this should be conducted disease by disease with specific pathologies characterizing each disease. Moreover, in the potpourri of conditions examined in this study, Behçet’s disease was included, which has no ICGA findings as the disease is limited to the retina. The study further reported a substantial proportion of eyes for which there were no corresponding OCT-A lesions because they were outside of the OCT-A captured area. Therefore, the conclusion that “the study demonstrates that OCT-A is a fast, non-invasive and high-resolution choroidal imaging technique to assess choriocapillaris and choroidal lesions in posterior uveitis” is misleading as the authors fail to add that the method has very limited applicability.

With the same aim of presenting OCT-A as a possible substitute for ICGA, another study on OCT-A in VKH was published with the title, “The Role of Optical Coherence Tomography Angiography in the Diagnosis and Management of Acute Vogt-Koyanagi-Harada Disease” [51] which makes the reader think that OCT-A has an established role in the diagnosis and management of VKH, which is far from being the case.

## 4. Discussion

OCT-A is a high-grade technological device allowing to establish intraocular vascular flow without having to use the injection of dyes. The non-invasive character of OCT-A has given rise to a great deal of hope that invasive techniques such as FA and ICGA might be replaced by OCT-A in uveitis. This was the paradigm followed by most of the OCT-A studies undertaken in the field of uveitis, in contrast to other new imaging modalities, such as OCT, EDI-OCT, and FAF, which provided useful additional information but did not claim to replace ocular angiography. 

Especially for those inflammatory conditions involving the retina, valuable findings were generated, such as a more precise evaluation of capillary damage in the posterior pole in different conditions, including HLA-A29 birdshot retinochoroiditis [3,42]. While such findings certainly represent an advancement in the research on disease mechanisms, they are far from serving the monitoring and management of these conditions. It is doubtful that the clinician can rely on OCT-A findings limited to a more or less limited area of the posterior pole while these conditions involve the posterior segment globally. Even if this were ultimately achieved with more performing instruments, the standardization of such findings would take a huge amount of data and time and would be hampered by the variety of instruments available. At best, OCT-A can give interesting additional elements that will, however, not be determined in decision making in the follow-up of patients. On the other hand, FA gives global information on retinal involvement, specifically on retinal vasculitis and exudation. The importance of vasculitis cannot be evaluated globally by OCT-A, even in wide-field devices such as the Canon Xephilio OCT-A, as it is unable to show leakage. For example, the profuse exudation which is present in the early stages of BRC occurs in the whole retina and is not taken into account by OCT-A, although this is a crucial element to be followed in order to judge the evolution of the disease and to orient therapeutical intervention [41].

In choriocapillaritis, OCT-A is able to show choriocapillaris drop-out but only in conditions where non-perfusion is more widespread due to the involvement of larger choriocapillaris vessels, as in severe MFC, APMPPE/AMIC or SC. Again, for these disorders, the exact localization and extent of lesions are accurately depicted by ICGA alone, which gives a global map of the lesions not limited to the posterior pole. Subsequently, indeed, the posterior pole drop-outs can be followed by OCT-A, and this avoids performing too many angiograms. However, the global evolution has still to be followed by ICGA, giving a picture of the whole fundus. In choriocapillaritis entities involving small distal end-capillaries, as in MEWDS, OCT-A is unable to detect whether there is non-perfusion or not. The flow in distal choriocapillaris is very reduced or absent as they behave like a sponge, and to identify these vessels, sufficient flow is not present. Therefore, some reports speak erroneously of an intact choriocapillaris in MEWDS. For these cases, the only means to show distal non-perfusion is ICGA. 

For stromal choroiditis, OCT-A is even less suited as it only shows the very diverse effects of choroidal infiltration on choroidal blood flow, a secondary effect of choroiditis difficult to interpret, as it can be due to choriocapillaris collapse due to choroidal granulomas or to choroidal atrophy. ICGA, in contrast, shows the primary lesion and choroidal infiltration and is very reactive to guide therapeutical intervention [52]. Moreover, as shown in Figure 4 and Figure 5, the bulk of lesions is in the periphery beyond the reach of today’s routine OCT-A instruments.

While ocular angiography (FA, ICGA), aided by BL-FAF and OCT, provides diagnostic clues for most of the uveitis entities and allows differential diagnosis of different etiologies, there is no evidence that OCT-A imaging can play such a role. OCT-A is unable to assess and follow posterior uveitis, for which it is not suited for the reasons given above. It should, therefore, not be advertised as a possible substitute for angiographic methods (FA and ICGA). However, OCT-A is an excellent imaging modality for research. As investigators usually closely work with technicians of OCT-A companies, they naturally tend to overestimate the value of OCT-A for routine follow-up purposes, which are commercially more interesting when having the status of a routine follow-up device rather than a device for research. 

In summary, OCT-A has been largely overestimated in the claim as a routine follow-up tool for the management of posterior uveitis and choroiditis, for which it is ill-suited.

## Figures and Tables

**Table 1:** Available OCT-A instruments on the market.

Model/Company	Scans/s	Technology	Field of View	Imaging Depth (mm)
ZEISS Angioplex™ OCT angiographic imaging on the CIRRUS™ [6]	68,000 A-scans/s	Full-spectrum	3 × 3 mm 6 × 6 mm 8 × 8 mm 12 × 12 mm	2.0–2.9
Optovue AngioVue^®^ (Optovue, Inc., Freemont, CA) [7]	70 000 A-scans/s	Split-spectrum	3 × 3 mm 6 × 6 mm 8 × 8 mm	2.0–3.0
Topcon^®^ Triton [8]	100,000 A-scans/s	Full-spectrum	3 × 3 mm 6 × 6 mm	2.6
Heidelberg engineering^®^ Spectralis OCT2 [9]	85,000 A-scans/s	Full-spectrum	3 × 3 mm 6 × 6 mm	2
Xephilio OCT-S1 Canon^®^	100,000 A-scans/s	Swept-source	3 × 3 mm 6 × 6 mm 8 × 8 mm 20 × 23 mm	5.3
RS-3000 Advance Angioscan (Nidek^®^)	53.000 A-scans/s	Spectral-domain	3 × 3 mm 6 × 6 mm 9 × 9 mm	2.5
Plex Elite 9000 Carl Zeiss Meditec^®^ *	Dual speed 100.000 and 200.000 A-scans/s	Swept-source	3 × 3 mm 6 × 6 mm 12 × 12 mm 9 × 15 mm	3.0–6.0

* Not commercialized, included in the Zeiss catalog as research OCT solutions.

## Data Availability

Not applicable.
